# Leupaxin Expression Is Dispensable for B Cell Immune Responses

**DOI:** 10.3389/fimmu.2020.00466

**Published:** 2020-03-25

**Authors:** Amélie Bonaud, Simon Clare, Valeria Bisio, John M. Sowerby, Shugang Yao, Hanne Ostergaard, Karl Balabanian, Kenneth G. C. Smith, Marion Espéli

**Affiliations:** ^1^Inflammation Chemokines and Immunopathology, Institut National de la Santé et de la Recherche Medicale (INSERM), Faculté de Médecine, Université Paris-Sud, Université Paris-Saclay, Clamart, France; ^2^Université de Paris, Institut de Recherche Saint Louis, EMiLy, Inserm U1160, Paris, France; ^3^Wellcome Trust Genome, Wellcome Trust Sanger Institute, Hinxton, United Kingdom; ^4^The Department of Medicine, Cambridge Biomedical, University of Cambridge School of Clinical Medicine, Cambridge, United Kingdom; ^5^Jeffrey Cheah Biomedical Centre Cambridge Biomedical, Cambridge Institute of Therapeutic Immunology & Infectious Disease, University of Cambridge, Cambridge, United Kingdom; ^6^Department of Medical Microbiology and Immunology, Li Ka Shing Institute of Virology, University of Alberta, Edmonton, AB, Canada

**Keywords:** leupaxin, B cells, plasma cells, cell activation, humoral immune response

## Abstract

The generation of a potent humoral immune response by B cells relies on the integration of signals induced by the B cell receptor, toll-like receptors and both negative and positive co-receptors. Several reports also suggest that integrin signaling plays an important role in this process. How integrin signaling is regulated in B cells is however still partially understood. Integrin activity and function are controlled by several mechanisms including regulation by molecular adaptors of the paxillin family. In B cells, Leupaxin (Lpxn) is the most expressed member of the family and *in vitro* studies suggest that it could dampen BCR signaling. Here, we report that *Lpxn* expression is increased in germinal center B cells compared to naïve B cells. Moreover, *Lpxn* deficiency leads to decreased B cell differentiation into plasma cells *in vitro*. However, Lpxn seems dispensable for the generation of a potent B cell immune response *in vivo*. Altogether our results suggest that Lpxn is dispensable for T-dependent and T-independent B cell immune responses.

## Introduction

B cells and plasma cells (PCs), corresponding to the terminal step of B cell differentiation, are key players of humoral immunity. After activation by antigen, B cells can either differentiate rapidly into PCs through the extrafollicular response or, following cooperation with T cells they can generate germinal centers (GC). In this structure B cells have an intense proliferative activity and are submitted to two genetic modification processes; somatic hypermutation (SHM) and class switch recombination (CSR) to improve respectively the affinity and effector properties of their B cell antigen receptor (BCR). The output of GCs is thus high-affinity and class-switched memory B cells or PCs (1). B cell activation, GC formation, high affinity B cell selection and PC differentiation rely on a tight regulation of the BCR signal strength (2–4). It is regulated by both positive and negative co-signaling through co-receptors including CD19 or CD22 for example (5–9). Integrins, although not co-receptors *per se*, play an important role in B cell activation. Integrins control B cell migration and adhesion (10–12) but their role is not limited to slowing down B cells. They also control the threshold of antigen-mediated signaling required for the full activation of B cells notably via the VLA4/VCAM1 complex (13).

Integrin activity and signaling are controlled by several mechanisms including regulation by the paxillin family of molecular adaptors (14). The paxillin family is composed of three members, Paxillin, Hic-5, and Leupaxin (Lpxn). This family presents a strong conservation with characteristic 3–5 amino terminal leucine-rich domain (LD) motifs and 4 Lin-11/Isl-1/Mec-3 (LIM) domains in C-terminus (C-ter) that are known to mediate protein-protein interactions (15). This family of molecular adaptors is mainly involved in the regulation of the dynamics of cellular adhesion *via* integrins. The two first members have been well-described and their function is better characterized than Lpxn (16). Indeed, Paxillin, and Hic5 were described in integrin-mediated focal adhesion points where they contribute to the recruitment of several proteins including MAPK leading to the reorganization of the actin cytoskeleton (17). Hic5 does not seem to be expressed in immune cells while Paxillin is mainly expressed by granulocytes (based on the ImmGen database). Lpxn expression was first reported in leukocytic cell lines (18) before being observed in cancerous tissues and osteoclasts (19, 20).

Lpxn seems to regulate integrin activity through the control of the phosphorylation of downstream adaptors. In the 293 cell line, Lpxn was shown to bind to PEST domain tyrosine phosphatase (PEP), an intracellular protein tyrosine phosphatase, implicated in the negative regulation of antigen receptor signaling in lymphocytes (21). Notably it was demonstrated that Lpxn via PEP participates to the dephosphorylation of Pyk2 and Fak that are key transducers of the intregrin-induced signaling cascade. Interestingly, Lpxn was also reported to interact directly with Pyk2 (18). Moreover, in A20 and cos1 cell lines and splenocytes, Lpxn inhibits the Ras pathway that is crucial during B cell activation (22, 23). Lpxn dysregulation was also shown to participate to tissue invasion by promoting cancerous cell adhesion and migration (19, 24). In addition, an integrin-independent role for Lpxn in BCR signaling was previously reported (25). In the human BJAB lymphoma cell line Lpxn interacts with Lyn at the plasma membrane upon BCR crosslinking. Moreover, Lpxn overexpression in the A20 B cell line decreased the BCR mediated phosphorylation of JNK and p38 MAPK (25). More recently a transcriptomic study suggested that Lpxn expression was under the control of the transcription factor IRF8 that itself negatively regulates GC B cells and PC differentiation (26). All these observations lead us to interrogate the role of Lpxn in B cell activation, GC formation and PC differentiation. Here, we report that *Lpxn* is more highly expressed in GC B cells than in naïve B cells. Moreover, using a total *Lpxn* deficient mouse model we showed that Lpxn is required for efficient PC differentiation *in vitro*. However, Lpxn seems to be dispensable for B cell activation *in vivo*.

## Materials and Methods

### Mouse Model and Immunization

The *Lpxn*^−/−^ mouse model was generated and provided by The Sanger Institute Mouse Resource (Cambridge, UK). All experiments were conducted in compliance with the European Union guide for the care and use of laboratory animals and has been reviewed and approved by an appropriate institutional review committee (C2EA-26, Animal Care and Use Committee, Villejuif, France) (approval number C9202301 project N°026). Immunization were performed intraperitoneally (ip) with 100 μg of 4-hydroxy-3-nitrophenylacetyl-keyhole limpet hemocyanine (NP-KLH) (Biosearch Technologies) adjuvanted with alum (Imject Alum, Thermo Scientific) for the first injection and with 100 μg NP-KLH in PBS for the second 28 days later or with 25 μg NP-LPS (Biosearch Technologies) in PBS. Blood samples for each point of the kinetic were obtained by submandibular puncture in accordance with maximum volume recommended by the local ethical rule. Blood samples at sacrifice were obtained by cardiac puncture immediately after euthanasia by inhalation of carbon dioxide.

### Cell Preparation and Flow Cytometry

Lymphoid organ cells [spleen, bone marrow, mesenteric lymph nodes (mLN), inguinal lymph nodes (iLN), or Peyer's patches (PPs)] were isolated and stained as previously described (27). Briefly, single cell suspensions were stained with appropriate antibodies (Supplementary Table 1) in PBS supplemented with 2% BSA and 2 mM EDTA for cell surface staining. Intracellular staining was performed using the Foxp3/Transcription factor staining buffer set (eBioscience) according to the provider recommendation. Flow cytometry analyses were performed on a BD LSR Fortessa cytometer and cell sorting experiments for qPCR analysis were performed using a BD FACS AriaII cell sorter. Data were analyzed with the Flowjo software (TreeStar, Ashland, OR). Splenic B cell magnetic enrichments for *in vitro* differentiation assay were performed using the CD43 depletion kit (Miltenyi Biotec) according to the manufacturer's recommendations.

### Western Blot Analysis

Splenocytes or the RAW264.7 cell line were resuspended in RIPA lysis buffer supplemented with Protease and Phosphatase Inhibitor (Thermofisher Scientific). Five or 30 μg of proteins were separated on a NuPAGE^TM^ 4–12% Bis-Tris Gel (Invitrogen) and transferred to a PVDF membrane. Primary antibodies against Lpxn (provided by HO), paxillin family protein (BD Biosciences) or β-actin (Cell Signaling) were incubated overnight at 4°C. Secondary antibodies, anti-rabbit IgG and anti-mouse IgG1, respectively (Jackson Immuno Research and Southern Biotech) conjugated to HRP were incubated 2 h at room temperature. Proteins were detected using Pierce ECL (Thermofisher Scientific) and signal was quantified by ChemiDoc™ Touch Gel Imaging System (BIO RAD). Band intensity was measured with ImageJ, background was subtracted, intensities were normalized to β-actin then to the WT control group.

### *In vitro* Cell Differentiation Assay

1 × 10^6^ splenocytes or 0,5 × 10^6^ B cells were stimulated with 5 μg/mL of lipopolysaccharide (LPS) (InVivoGen) with or without 20 ng/mL of IL-4 (Miltenyi) or with 5 μg/mL anti-CD40 antibody (R&Dsystems) and 20 ng/mL of IL-4 for 3 days in RPMI supplemented with 10% fetal calf serum, 0.05 mM 2-mercaptoethanol, 100 U/mL penicillin-streptomycin, 1 mM sodium pyruvate, and non-essential amino acids as recommended (Gibco). Supernatants were used for ELISA quantification assay and cells were analyzed by flow cytometry at day 3.

### Enzyme-linked Immunosorbent Assay (ELISA) and ELISpot

ELISA and ELISpot assays were performed as previously described (28) for the determination of NP-specific antibody titres and for the quantification of NP-specific antibody secreting cells (ASCs), respectively. ELISA was also used for the detection of total IgM secreted in culture supernatant. Briefly, plates were pre-coated with goat anti-mouse IgM (Southern Biotech) or with NP (4)-BSA/NP (15)-BSA (Biosearch Technologies). After a step of saturation, 5 × 10^5^ cells per well for ELISpot or diluted sera/supernatants for ELISA were added before staining the fixed antibodies with a peroxidase-conjugated secondary antibody. Enzymatic revelation was performed with AEC (Sigma-Aldrich) for the ELISpot and with the TMB substrate reagent set (BD OptEIA) for ELISA. All Antibodies and the concentration used are indicated in Supplementary Table 1.

### Quantitative PCR

Total RNA was extracted using the RNeasy Mini kit (Qiagen). Reverse transcription was performed using M-MLV reverse transcriptase (Invitrogen) with (dT)_25_ primer (Invitrogen) according to the supplier's recommendations. Relative quantification was performed with LightCycler Taqman Master (Roche) on cDNA samples (20 ng per reaction). Quantification of the gene of interest was performed as previously described (29) by the ΔCt method with the mean of *Gapdh* and *Actin b* used as housekeeper genes. TaqMan probes for *Gapdh* (Mm99999915_g1), *Actin b* (Mm01205647_g), *Aicda* (Mm01184115_m1), and *Lpxn* (Mm00547686_m1) (Applied Biosystems) were used.

## Results

### Lpxn Is Highly Expressed in Germinal Center B Cells

Publicly available transcriptomic datasets suggest that, among the B cell lineage, Lpxn is particularly highly expressed in germinal center (GC) B cells (Supplementary Figure 1A). To confirm this observation we sorted naïve B cells (B220^+^CD19^+^/ FAS^lo^GL7^lo^) and GC B cells (B220^+^CD19^+^/FAS^hi^GL7^hi^) and analyzed the expression of *Lpxn* on these two cell subsets as well as on total splenocytes. The result obtained supports that indeed *Lpxn* expression in GC B cells is at least 4 times higher than in naïve B cells and 7 times higher than in total splenocytes (Supplementary Figure 1B). As a positive control, we also measured the expression of *Aicda* that is increased in GC B cells compared to naïve B cells (30). In order to characterize whether Lpxn plays a role in B cell activation and subsequently on GC B cell biology we obtained a *Lpxn* deficient mouse model created by the Sanger Institute Mouse Resource via a “knock-out first” approach leading to the generation of a total knock-out (31) (Supplementary Figure 2A). *Lpxn* transcript expression was measured by qPCR on B cells from *Lpxn*^+/+^, *Lpxn*^+/−^, and *Lpxn*^−/−^ mice (Supplementary Figure 2B) confirming the total loss of expression of *Lpxn* in *Lpxn*^−/−^ mice. Interestingly, we observed an allele-dose dependent decrease in the expression of *Lpxn*. Furthermore, we confirmed by western blot the allele-dose dependent loss of Lpxn expression in *Lpxn*^−/−^ mice (Supplementary Figures 2C–E). In addition, Lpxn deficiency was not associated with compensatory expression of Paxillin or Hic-5 (Supplementary Figure 2E).

*Lpxn* deficiency did not affect spleen, Peyer's patches (PP), mesenteric lymph nodes (mLN), inguinal lymph nodes (iLN) and bone marrow (BM) cellularity (Supplementary Table 2). Moreover, the percentage of BM precursor B cells, immature B cells, mature B cells and transitional B cells were comparable between *Lpxn*^+/+^, *Lpxn*^+/−^, and *Lpxn*^−/−^ mice (Table 1). In the spleen, the frequency of marginal zone B cells, follicular B cells and B1 cells of the three genotypes were also similar suggesting that Lpxn is not necessary for B cell development (Table 1 and Supplementary Figure 3 for the gating strategy).

**Table 1:** Lpxn expression does not impact B cell ontogeny.

		***Lpxn^**+/+**^***	***Lpxn^**+/−**^***	***Lpxn^**−/−**^***
BM	Pre- and pro-B cells	17.79 ± 3.38	15.56 ± 3.41	16.86 ± 2.39
	Immature B cells	4.08 ± 0.74	3.10 ± 0.79	3.77 ± 0.74
	Transitional B cells	0.16 ± 0.03	0.11 ± 0.04	0.12 ± 0.03
	Mature B cells	5.61 ± 1.15	4.94 ± 1.17	4.82 ± 0.93
Spleen	MZ B cells	7.4 ± 1.52	9.09 ± 1.49	6.67 ± 1.34
	Follicular B cells	55.17 ± 0.68	53.37 ± 3.14	56.10 ± 4.31
	B1a B cells	0.06 ± 0.008	0.05 ± 0.003	0.05 ± 0.01
	B1b B cells	0.92 ± 0.034	1.02 ± 0.07	1.04 ± 0.10

*The frequency of each B cell subset in the BM and spleen of Lpxn^+/+^, Lpxn^+/−^ or Lpxn^−/−^ mice was determined by flow cytometry. BM B cell subsets were gated as: B220^low^IgM^−^ (pre- and pro-B cells), B220^low^IgM^low/+^ (Immature B cells), B220^+^IgM^low/+^ (Mature B cells), and B220^low/+^IgM^+^ (Transitional B cells). Splenic B cell subsets were gated as: B220^+^CD21^low^CD23^high^ (Follicular B cells) and B220^+^CD21^high^CD23^low^ [Marginal zone B cells (MZ)], CD19^+^B220^−^CD11b^+^CD5^+^ (B1a B cells), and CD19^+^B220^−^CD11b^+^CD5^−^ (B1b cells). The mean +/– the standard error of the mean (sem) are shown. N = 3 mice in at least three independent experiments. We did not observe significant difference between the three genotypes with the two-tailed Mann-Whitney non-parametric test. The gating strategies are shown in Supplementary Figure 3*.

### Lpxn Expression Is Required for Plasma Cell Differentiation *in vitro*

**Figure 1:**
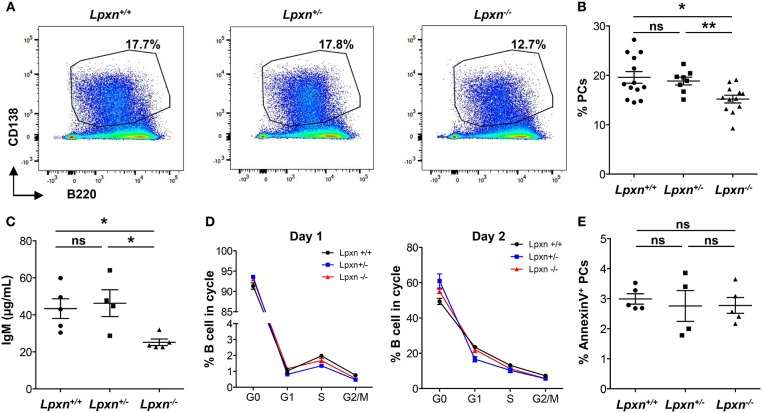
Lpxn promotes PC differentiation *in vitro*. **(A)** Representative dot plots for PCs (gated as CD138^high^B220^low^) generated from *Lpxn*^+/+^, *Lpxn*^+/−^, and *Lpxn*^−/−^ splenocytes after 3 days of stimulation with LPS. **(B)** Quantification of the frequency of PCs generated from *Lpxn*^+/+^, *Lpxn*^+/−^, and *Lpxn*^−/−^ splenocytes after 3 days of stimulation with LPS. **(C)** ELISA quantification of total IgM secreted in the culture supernatant after 3 days of LPS stimulation. **(D)** The cell cycle status of stimulated B cells was determined by flow cytometry. G0, G1, S, and G2/M cells were gated as DAPI^−^Ki-67^−^, DAPI^−^Ki-67^+^, DAPI^int^Ki-67^+^, and DAPI^+^Ki-67^+^, respectively. *Lpxn*^+/+^, *Lpxn*^+/−^, and *Lpxn*^−/−^ mice are represented by black, blue and red lines, respectively. **(E)** The frequency of apoptotic PCs at day 3 post LPS stimulation was determined by flow cytometry with an AnnexinV staining. For flow cytometry experiment cells were gated on their size and structure, on their viability (Live/Dead zombie aqua staining) and doublets were excluded. *N* = 3–5 mice in at least three independent experiments. For **(B)**, three experiments were pooled. For **(B,C,E)** each symbol represents an individual mouse with the mean and the sem also represented. The *p*-values were determined with the two-tailed Mann-Whitney non-parametric test. *p < 0.05; **p < 0.01. “ns” = non-significant *p*-value.

We next wanted to assess whether *Lpxn* deficiency affects B cell activation and differentiation. For this we first analyzed PC differentiation *in vitro* following B cell activation with LPS. After 3 days of stimulation we observed that *Lpxn*^−/−^ B cells differentiated significantly less into PCs than their WT and heterozygous counterparts (Figures 1A,B). This was also observed when sorted B cells were used for *in vitro* differentiation (Supplementary Figures 4A,B). Moreover, this decreased PC differentiation was associated with decreased IgM production (Figure 1C). This decrease was not due to a defect in B cell cycling as shown by analyzing cell cycle with a Ki-67/DAPI staining after 1 and 2 days of stimulation (Figure 1D). PC survival was not affected either by *Lpxn* deficiency (Figure 1E). Altogether, these results suggest that Lpxn is important for B cell differentiation into PCs upon LPS stimulation but not for B cell proliferation or PC survival. Strikingly, although they express significantly less *Lpxn* than the wt mice, *Lpxn*^+/−^ mice did not present an alteration of PC differentiation suggesting the existence of a threshold for Lpxn expression above which PC differentiation occurs normally.

We next wondered whether Lpxn also affects B cell differentiation following a T-dependent activation mimicked *in vitro* through stimulation with an anti-CD40 Ab. Following stimulation, however, PC differentiation was comparable between the three groups of mice (Supplementary Figure 5A). We also assessed whether Lpxn may be important for class-switch recombination (CSR) by assessing CSR to the IgG1 isotype after anti-CD40/IL-4 and LPS/IL-4 stimulation for 3 days. As shown in Supplementary Figures 5B,C, the frequency of IgG1^+^ B cells was similar between *Lpxn*^+/+^, *Lpxn*^+/−^, and *Lpxn*^−/−^ mice in both conditions suggesting that Lpxn is dispensable for IgG1 CSR.

### Lpxn Is Dispensable for Germinal Center and Plasma Cell Generation at Steady State

**Figure 2:**
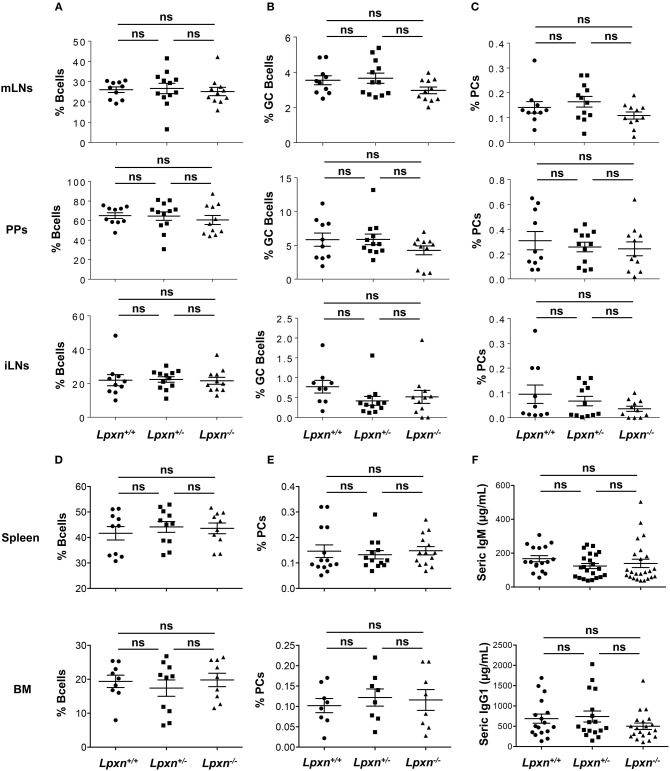
Lpxn is dispensable for germinal center and plasma cell generation at steady state. **(A–E)** Quantification of the frequency of B cells **(A)**, GC B cells **(B)**, and PCs **(C)** in mesenteric lymph nodes (mLNs) (top row), Peyer's patches (PPs) (second row), and inguinal LNs (iLNs) (third row), and frequency of B cells **(D)**, and PCs **(E)** in spleen (fourth row), and bone marrow (bottom row) at steady state. By flow cytometry B cells were gated as B220^+^, GC B cells as B220^+^GL7^+^Fas^+^ and PCs as B220^low/−^CD138^+^. Cells were gated on their size and structure, on their viability (Live/Dead zombie aqua) and doublets were excluded. **(F)** ELISA quantification of seric IgM (top) and IgG1 (bottom) in *Lpxn*^+/+^, *Lpxn*^+/−^, and *Lpxn*^−/−^ mice at steady state. For all panels each symbol represents an individual mouse with the mean and the sem also represented. Two or three experiments were pooled. The p-values were determined with the two-tailed Mann-Whitney non-parametric test. All *p*-values were non significant (“ns”).

Considering the increased expression of *Lpxn* in GC B cells and the impact of *Lpxn* deficiency on B cell activation and PC differentiation we next analyzed GC B cell and PC frequency in the primary and secondary lymphoid organs of *Lpxn*^+/+^, *Lpxn*^+/−^, and *Lpxn*^−/−^ mice at steady state. B cell frequencies in iLNs, mLNs, PPs, spleen, and BM were not affected by *Lpxn* deficiency (Figures 2A,D). We observed a slight but non-significant decrease in the frequency of GC B cells in both mLNs and PPs of *Lpxn*^−/−^ mice compared to *Lpxn*^+/−^ and *Lpxn*^+/+^ mice (Figure 2B). In parallel, we assessed T cell help and showed that the frequency of Th2- (Gata3^+^), Th17- (RORγT^+^) and Th1- (Tbet^+^) –biased T helper cells were equivalent in *Lpxn*^+/+^, *Lpxn*^+/−^, and *Lpxn*^−/−^ mice (Supplementary Figure 6). Moreover, PC frequency was not affected by *Lpxn* deficiency in any of the 5 tissues analyzed (Figures 2C,E). Supporting this data, we observed that baseline titres of circulating IgM and IgG1 were similar between the three experimental groups (Figure 2F). Thus, at steady state *Lpxn* expression does not seem essential for spontaneous GC formation nor PC development.

### Lpxn Is Not Essential for Plasma Cell Generation After a T-Dependent Immunization

**Figure 3:**
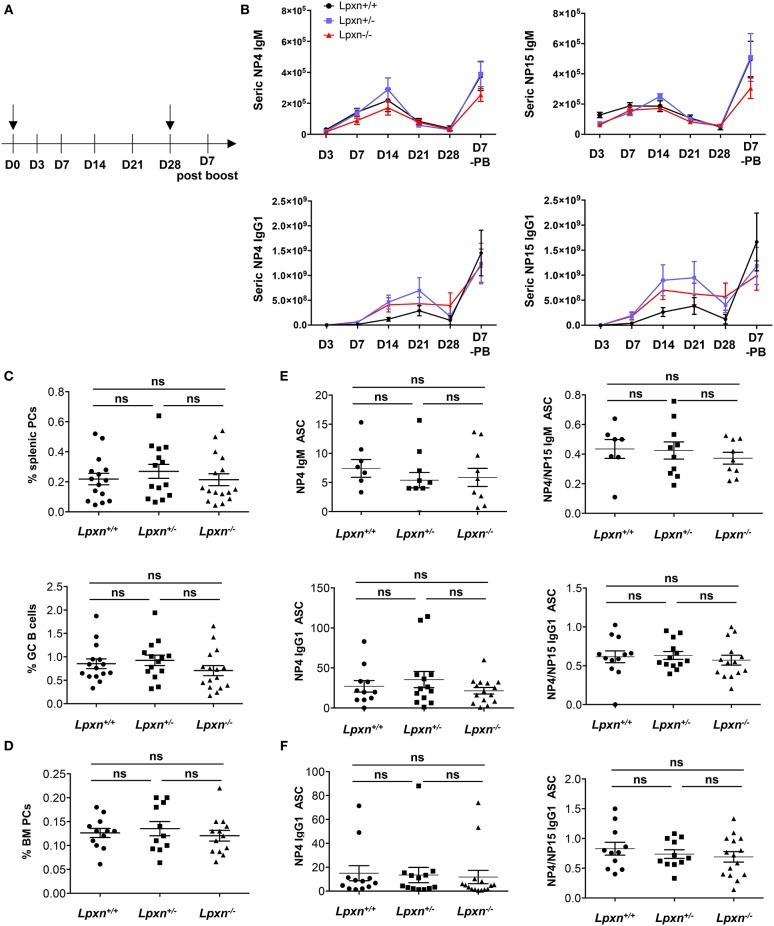
Lpxn is not essential for plasma cell generation after a T-dependent immunization. **(A)** Schematic representation of the immunization protocol. NP-KLH injections are indicated by black arrow and bleeding is indicated by lines. **(B)** ELISA quantification of seric NP4- (left panel) and NP15- (right panel) specific IgM and IgG1 at each point in *Lpxn*^+/+^ (black line), *Lpxn*^+/−^ (blue line), and *Lpxn*^−/−^ (red line) mice. **(C)** Frequency of splenic PCs (B220^low/−^CD138^+^, top) and GC B cells (B220^+^GL7^+^Fas^+^, bottom) determined by flow cytometry 7 days after boost. **(D)** Frequency of BM PCs (B220^low/−^CD138^+^) determined by flow cytometry 7 days after boost. Cells were gated on their size and structure, on their viability (Live/Dead zombie aqua) and doublets were excluded. **(E,F)** Quantification of NP-specific IgM and IgG1 ASCs from the spleen **(E)** and the BM **(F)** by ELISPOT. The affinity maturation was determined by calculating the ratio of NP4/NP15 ASCs in each condition (right panels). Four independent experiments were pooled. The *p*-values were determined with the two-tailed Mann-Whitney non-parametric test. All *p*-values were non significant (“ns”).

To assess more precisely the impact of Lpxn on GC B cell and PC differentiation *in vivo* we performed immunization with the well-characterized T-dependent antigen NP-KLH to induce a potent GC-mediated immune response in the spleen (27, 32). Mice were immunized ip with NP-KLH in alum at d0, boosted with NP-KLH only at day 28 and bled at day 3, 7, 14, 21, 28, and at sacrifice (Figure 3A). By ELISA, we observed a normal kinetic of the immune response with a peak of Ig secreted between day 14 and 21 post primary immunization and a robust increase 7 days after the boost immunization in the three experimental groups. Moreover, the level of NP-specific IgM and IgG1 were similar for *Lpxn*^+/+^, *Lpxn*^+/−^, and *Lpxn*^−/−^ mice (Figure 3B, right). We also assessed the quality of the response by measuring high affinity NP-specific IgG (NP4 binding) and we did not observe difference between the three groups (Figure 3B, left). At day 7 post boost we analyzed by flow cytometry the percentage of GC B cells and PCs in the spleen and BM of immunized animals. In agreement with the ELISA data, the three experimental groups displayed equivalent frequencies of total GC B cells and PCs in the spleen and BM (Figures 3C,D and Supplementary Figure 7). Finally, using ELISPOT, we analyzed at day 7 post boost NP-specific Ab secreting cells (ASCs) in the spleen and BM (Figures 3E,F, respectively). Again, we did not detect any difference between the 3 genotypes in terms of NP-specific ASC frequency (Figures 3E,F, left) nor affinity maturation as assessed by the NP4/NP15 ratio (Figures 3E,F, right). In addition, a similar result was obtained after sheep red blood cell immunization (data not shown). Altogether, our *in vivo* data suggest that Lpxn is dispensable for GC formation and PC differentiation after a T-dependent immunization.

### Lpxn Is Dispensable for T-independent Plasma Cell Generation

**Figure 4:**
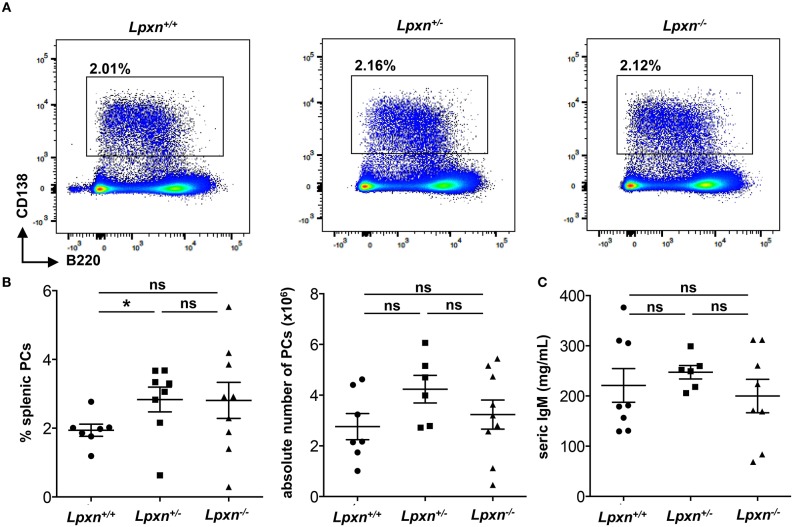
Lpxn is dispensable for T-independent plasma cell generation. **(A)** Representative dot plot of splenic PCs gated as B220^low/−^CD138^+^ 3 days after NP-LPS immunization. **(B)** Percentage and absolute number (left and right, respectively) of splenic PCs determined by flow cytometry 3 days after NP-LPS immunization. Cells were gated on their size and structure, on their viability (Live/Dead zombie aqua) and doublets were excluded. **(C)** ELISA quantification of seric IgM at D3 after NP-LPS immunization. Two independent experiments were pooled. The *p*-values were determined with the two-tailed Mann-Whitney non-parametric test. *p < 0.05; “ns” = non-significant *p*-value.

As we observed a decreased PC differentiation *in vitro* after LPS stimulation, we wondered if such a defect could be observed in the frame of a T-independent immune response. To verify this possibility we performed T-independent immunization with NP-LPS and analyzed the generation of PCs 3 days later. By flow cytometry we observed no difference in the frequency or number of splenic PC between *Lpxn*^+/+^, *Lpxn*^+/−^, or *Lpxn*^−/−^ mice (Figures 4A,B). Moreover, the titres of secreted IgM were equivalent in the sera of mice from the 3 genotypes (Figure 4C). Thus, despite its clear impact *in vitro*, Lpxn appears not essential for the development of a potent T-independent immune response *in vivo*.

## Discussion

B cell activation relies on the integration of several signals including BCR-antigen interaction and integrin-mediated adhesion and signaling. Multiple reports in the literature hint at a potential role for Lpxn in this process. Using a *Lpxn* full knock-out mouse model we thoroughly investigated if this was the case. Our results demonstrate that Lpxn is required for efficient PC differentiation *in vitro* upon LPS stimulation. Of note the gene dose effect observed for *Lpxn* expression in *Lpxn*^+/+^, *Lpxn*^+/−^, and *Lpxn*^−/−^ mice does not translate into differential PC differentiation. In fact low level of Lpxn expression, as observed in heterozygous mice, seems sufficient to maintain a potent PC differentiation. However, immunization-induced T-dependent and T-independent humoral responses were normal in absence of Lpxn suggesting that this protein is dispensable. In particular, we carefully analyzed GC B cells. We initially showed that *Lpxn* is highly expressed in GC B cells while the two other members of the family, Paxillin and Hic-5 are barely detectable in this cell subset (as reported by the ImmGen Consortium) (33). However, the kinetic and depth of the GC B cell response was not affected by *Lpxn* deficiency. We next wondered if the quality of this response might be altered. We observed that Lpxn is dispensable for proper affinity maturation. Finally, we also showed that in absence of Lpxn PC generation was not affected after both T-dependent and T-independent immunizations. The discrepancy observed between our *in vitro* and *in vivo* results could have several causes. First, Lpxn deficiency may impact differently different cell types. Indeed we used a total Lpxn KO mouse model and we cannot rule out a compensatory mechanism driven by the splenic environment and not recapitulated in *in vitro* B cell cultures. The generation of a B-cell specific conditional KO model could be interesting to assess this point. Another possibility could be that other members of the paxillin family compensate for Lpxn deficiency *in vivo*. Indeed, although both Lpxn and Paxillin regulate integrin-mediated cell adhesion and focal adhesion, they appear to have distinct but interwoven roles (34). A direct link may exist between Paxillin and Lpxn, with Lpxn acting as a negative regulator of Paxillin in integrin-mediated cell adhesion (35). Moreover, Hic5 is able to compensate for the lack of Paxillin (36). We assessed the protein expression of *Paxillin* and *Hic5* in B cells from *Lpxn*^+/+^*, Lpxn*^+/−^, and *Lpxn*^−/−^ mice and observed that Hic5 was weakly expressed as suggested by previous reports (ImmGen consortium) and that their expression was not significantly affected by the loss of Lpxn (Supplementary Figure 2E). Further work would be required to determine whether Paxillin is sufficient to compensate for Lpxn deficiency. Our results thus suggest that Lpxn is not essential for B cell activation *in vivo* and that integrin regulation in this cell type is likely to be independent of this molecule. Whether other molecular mechanisms can compensate for Lpxn deficiency will require further investigation. Taken together, our results support a role for Lpxn in the generation of PCs but some compensatory mechanisms are likely to occur *in vivo*.

## Data Availability Statement

The raw data supporting the conclusions of this article will be made available by the authors, without undue reservation, to any qualified researcher.

## Ethics Statement

The animal study was reviewed and approved by C2EA-26, Animal Care and Use Committee, Villejuif, France.

## Author Contributions

AB designed the study, performed and analyzed experiments, and wrote the manuscript. SC provided the mouse model. VB and JS performed experiments. SY and HO provided a critical reagent. KS contributed to the study design. KB reviewed the manuscript. ME designed the study, performed and analyzed experiments, and wrote the manuscript.

### Conflict of Interest

The authors declare that the research was conducted in the absence of any commercial or financial relationships that could be construed as a potential conflict of interest.
