# Supplementary material for: Leupaxin Expression Is Dispensable for B Cell Immune Responses
Source: Front Immunol. 2020 Mar 25;11:466. doi: 10.3389/fimmu.2020.00466 (PMC7109257; doi:10.3389/fimmu.2020.00466)
Supplement: Supplementary file 1 [file Data_Sheet_1.pdf]

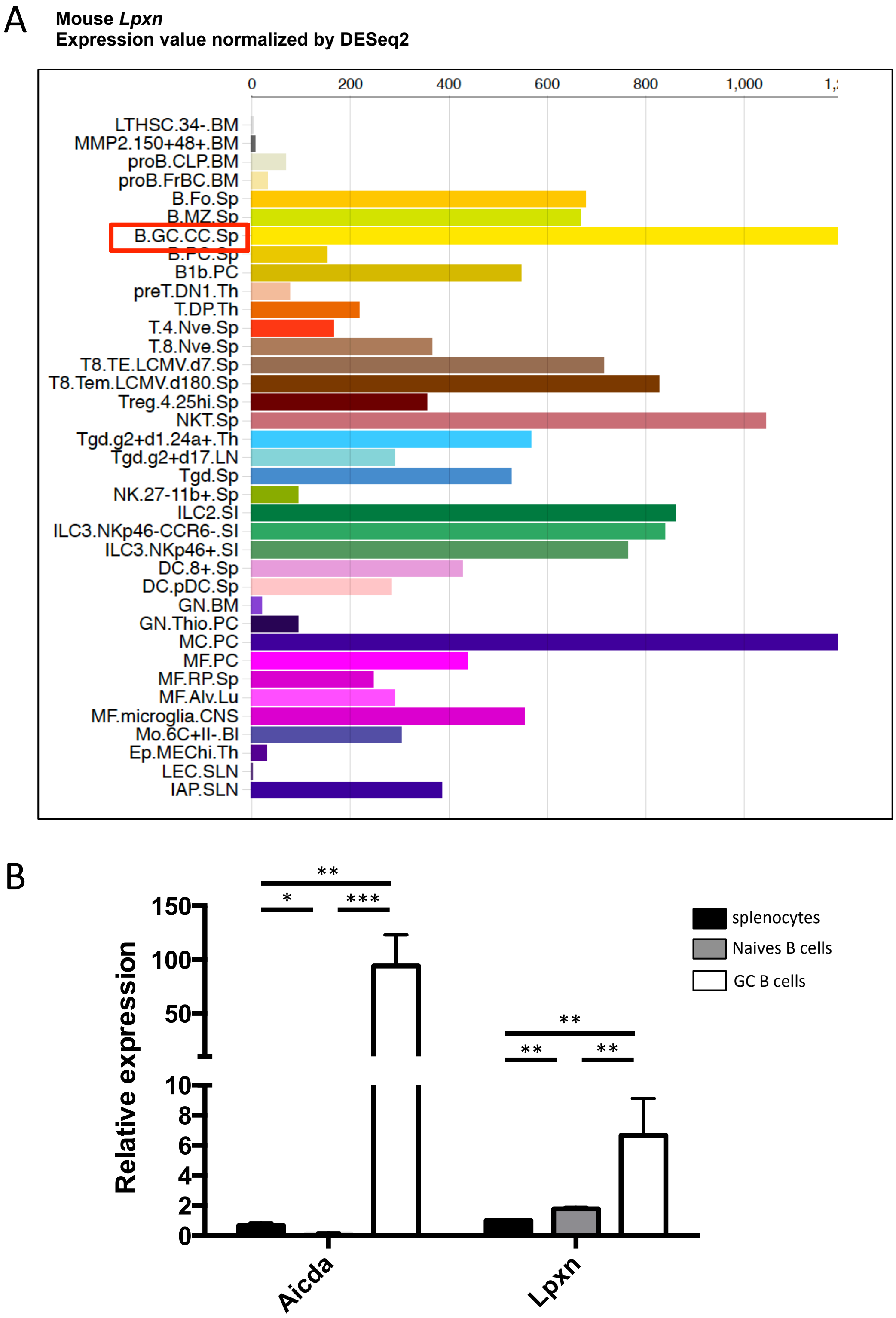

Supplementary Figure 1: ***Lpxn* expression in the B cell compartment.** (A) Relative abundance of *Lpxn* expression in immune lineage cell subsets. Data from www.immgen.org database. (B) Relative expression of *Aicda* and *Lpxn* in splenocytes (black), naive B cells (grey) or germinal center B cells (white) from Peyer's patches. N=6 mice in 2 experiment. The p-values were determined with the two-tailed Mann-Whitney non parametric test. *: p<0,05; **: p<0,01 or "ns" when p-values were not significant.

Supplementary Figure 1

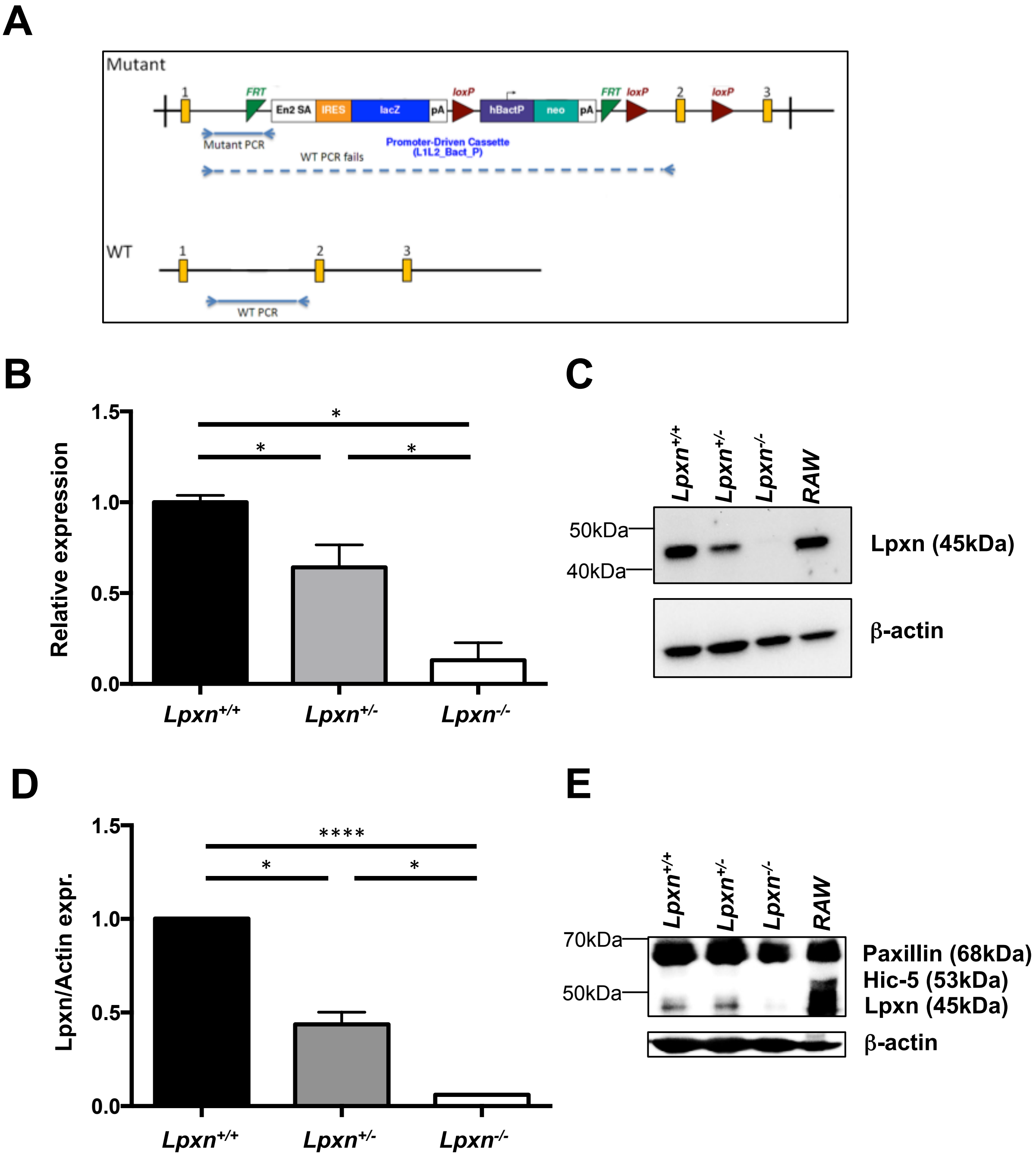

Supplementary Figure 2: ***Lpxn* KO mouse model**. (A) Schematic representation of the *Lpxn* locus and targeting strategy for the generation of the *Lpxn* KO mouse model. Primer binding sites for the genotyping of the animals are indicated (blue arrows). (B) Relative expression of *Lpxn* transcripts in B cells from *Lpxn*$^{+/+}$, *Lpxn*$^{+/-}$ and *Lpxn*$^{-/-}$ mice . N=4 mice. (C) Western blot analysis of Lpxn (top) and β-actin (bottom) in splenic cells from *Lpxn*$^{+/+}$, *Lpxn*$^{+/-}$ and *Lpxn*$^{-/-}$ mice as well as in the RAW264.7 cell line as positive control. (D) Relative expression of Lpxn normalized to the *Lpxn*$^{+/+}$ group. Band intensity was measured with ImageJ, background was subtracted, intensities were normalized to β-actin then to the WT control group (n=2). (E) Western blot analysis of the paxillin family members (Paxillin, Hic-5 and Lpxn) (top) and β-actin (bottom) in splenic cells from *Lpxn*$^{+/+}$, *Lpxn*$^{+/-}$ and *Lpxn*$^{-/-}$ mice as well as in the RAW264.7 cell line as positive control. The p-values were determined with the two tailed Mann-Whitney non parametric test (B) or with unpaired t test (D) . *: p<0,05; ****: p<0,0001.

Supplementary Figure 2

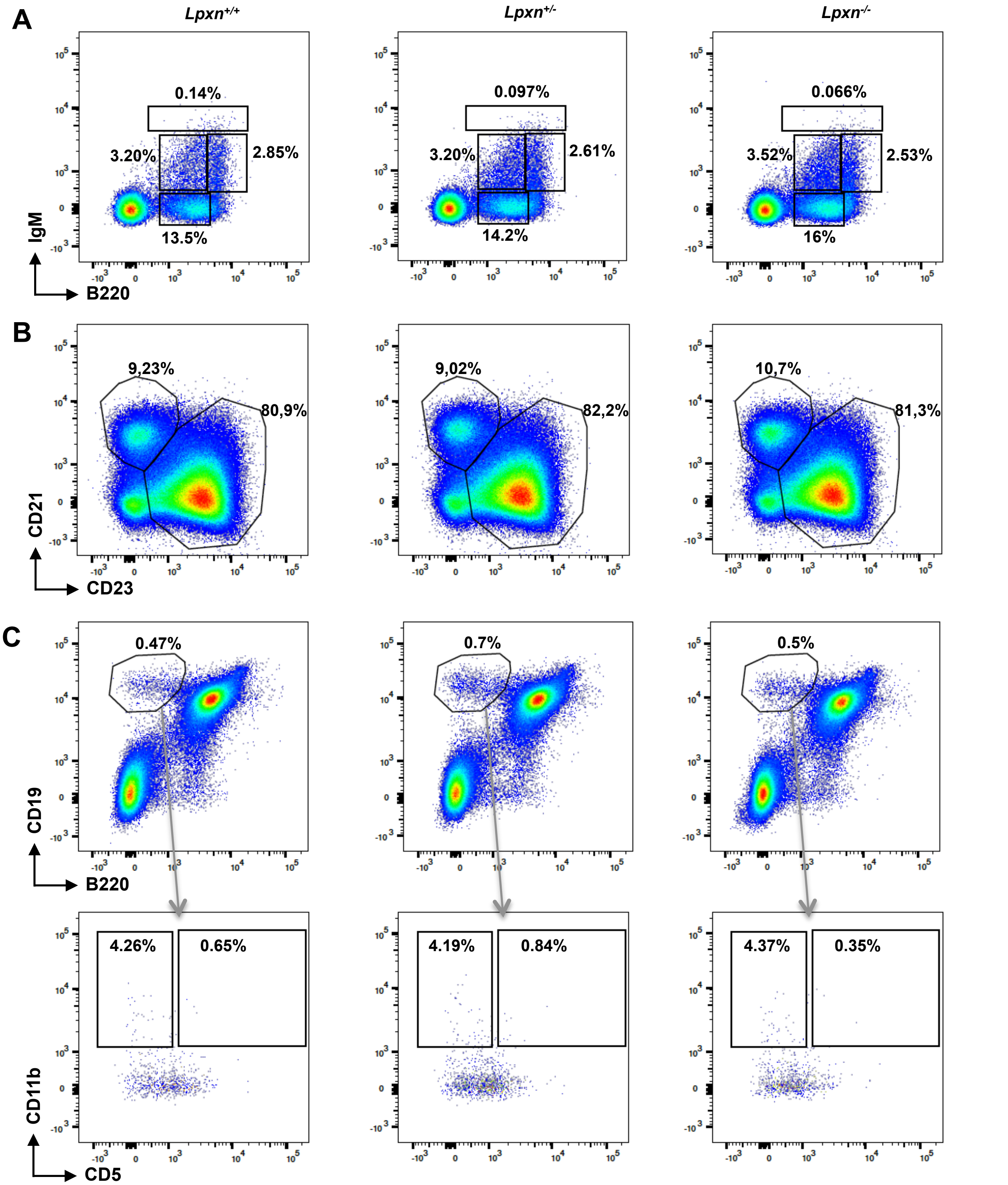

Supplementary Figure 3: **Gating strategy for spleen and bone marrow cells**. (A) Gating strategy for the identification of B lineage cell subsets in the bone marrow of *Lpxn*$^{+/+}$ (left), *Lpxn*$^{+/-}$ (middle) or *Lpxn*$^{-/-}$ (right) mice determined by flow cytometry. Bone marrow B-lineage cells were defined as B220^{low}IgM^{-} (precursors), B220^{low} IgM$^{low/+}$ (immature), B220^{+} IgM$^{low/+}$ (mature) and B220$^{low/+}$ IgM^{+} (transitional). (B) Gating strategy for the identification of follicular and marginal zone B cells in the spleen of *Lpxn*$^{+/+}$, *Lpxn*$^{+/-}$ or *Lpxn*$^{-/-}$ mice. B cells were first gated as B220^{+}CD19^{+} and follicular and marginal zone B cells were respectively gated as CD21$^{+/-}$CD23^{+} and CD21^{+}CD23^{low}. (C) Gating strategy for the identification of B1a and B1b cells in the spleen of *Lpxn*$^{+/+}$, *Lpxn*$^{+/-}$ or *Lpxn*$^{-/-}$ mice. B1 cells were first gated as B220^{-}CD19^{+} (top row) and then as CD11b^{+}CD5^{-} for B1b and CD11b^{+}CD5^{-} for B1a cells. For all gating strategies cells were first gated on their size and structure, their viability (Live/ Dead zombie aqua) and doublets were excluded.

Supplementary Figure 3

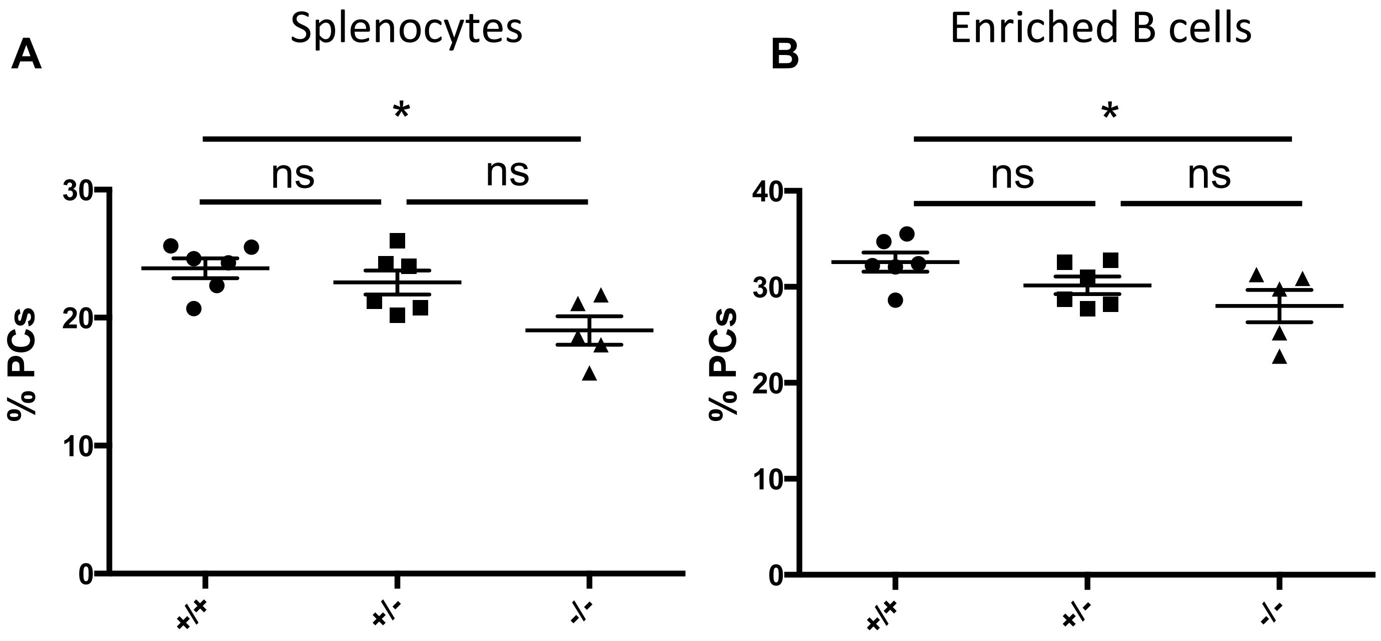

Supplementary figure 4: **Plasmablast differentiation upon LPS stimulation of splenocytes or purified B cells**. Percentage of plasmablasts (B220$^{+/low}$ CD138^{+}) were determined after 3 days of LPS stimulation of splenocytes (A) or enriched B cells (CD43 negative cell enrichment using beads from Miltenyi Biotech) (B) of *Lpxn*$^{+/+}$, *Lpxn*$^{+/-}$ and *Lpxn*$^{-/-}$ mice. Cells were first gated on their size and structure, their viability (Live/Dead zombie aqua) and doublets were excluded. N= 5-6 mice. The p-values were determined with the two-tailed Mann Whitney non parametric test. *: $p<0.05$ or "ns" when p-values were not significant.

Supplementary Figure 4

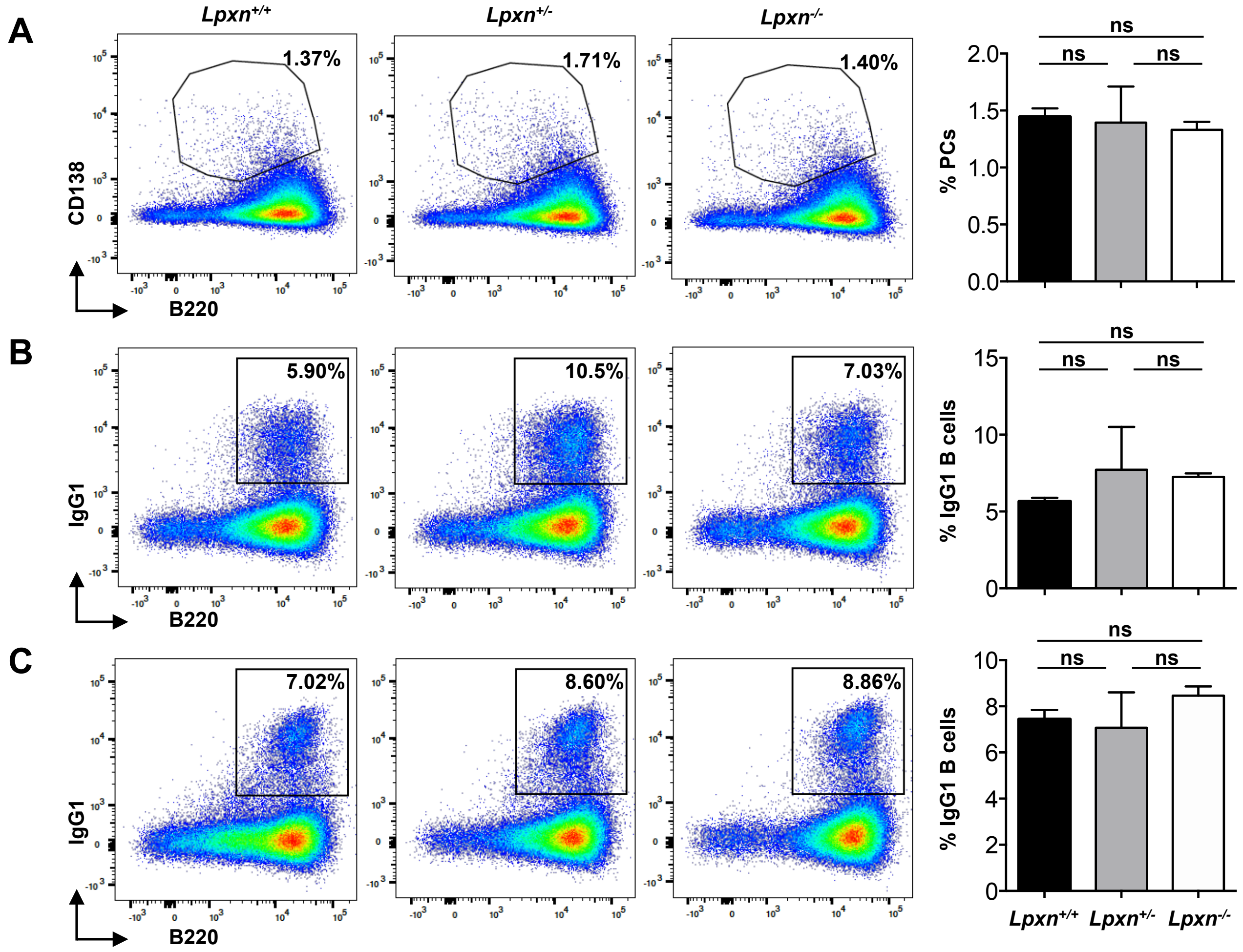

Supplementary Figure 5: **Analysis of Ig switching in the Lpxn KO mouse model *in vitro*.** B cells were stimulated with CD40-Il4 (A-B) or LPS-Il4 (C) during 3 days and analysed by flow cytometry. (A) Representative gating and percentage of plasmablasts ($B220^{+/low}$ $CD138^{+}$) are indicated. (B-C) Representative gating and percentage of $IgG1^{+}$ B cells ($B220^{+}$ $IgG1^{+}$) are indicated. Histograms at the end of each line represent the percentage of plasmablasts (A) or $IgG1^{+}$ B cells (B-C). Cells were first gated on their size and structure, their viability (Live/Dead zombie aqua) and doublets were excluded. N= 6 mice from 3 independent experiments. The p-values were determined with the two-tailed Mann-Whitney non parametric test. All p-values were not significant (“ns”).

Supplementary Figure 5

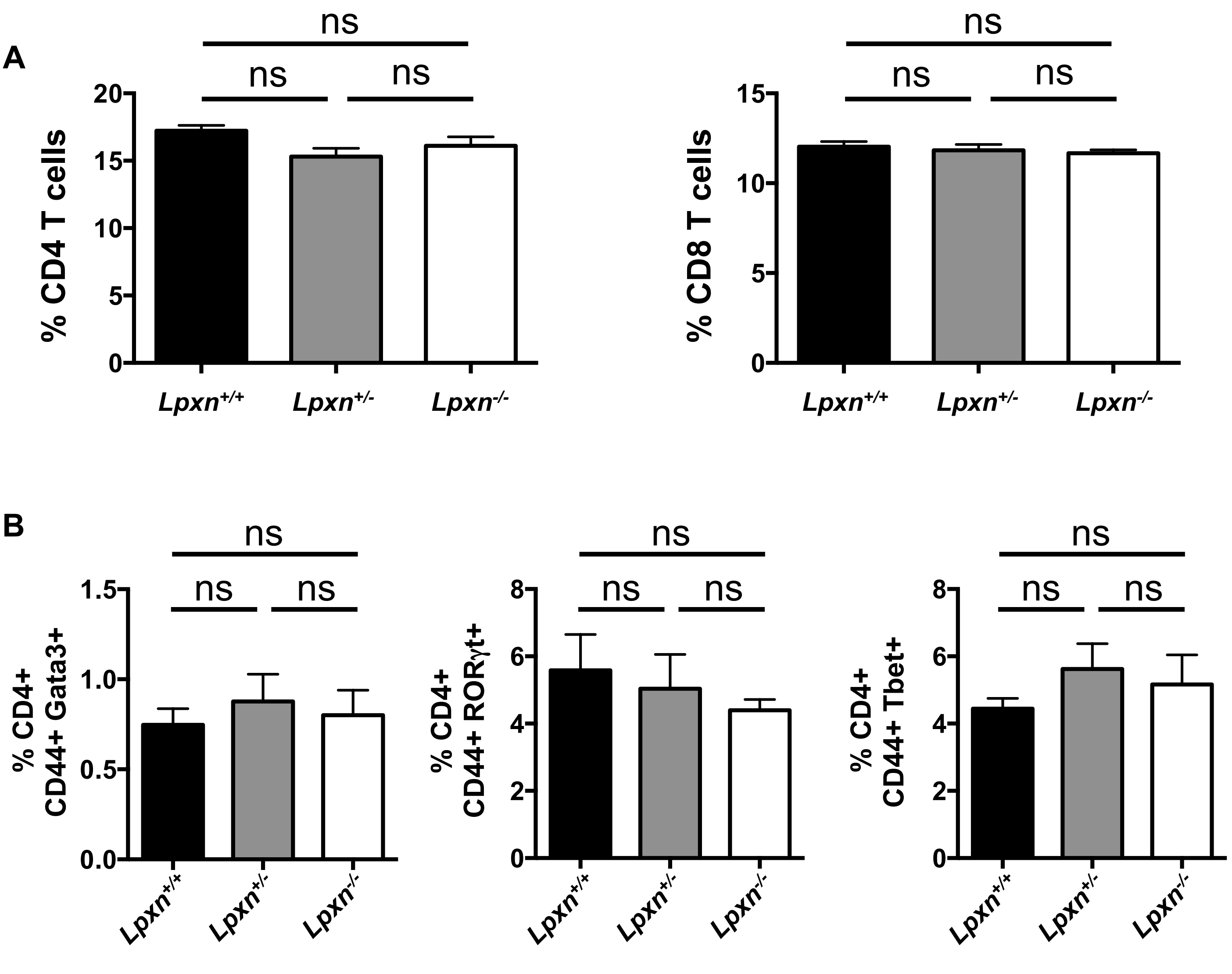

Supplementary Figure 6: **Analysis of the T cell splenic compartment**. (A) Percentage of $CD4^+$ or $CD8^+$ T cells in the spleen of *Lpxn*$^{+/+}$ (black), *Lpxn*$^{+/-}$ (grey) and *Lpxn*$^{-/-}$ (white) mice. (B) $CD4^+$ $CD44^+$ T cell subsets were analysed in function of their expression of Gata3 (left) RORγt (middle) or Tbet (right). Cells were gated on their size and structure, their viability (Live/Dead zombie aqua) and doublets were excluded. N= 5-6 mice. The p-values were determined with the two-tailed Mann Whitney non parametric test. All p-values were not significant ("ns").

Supplementary Figure 6

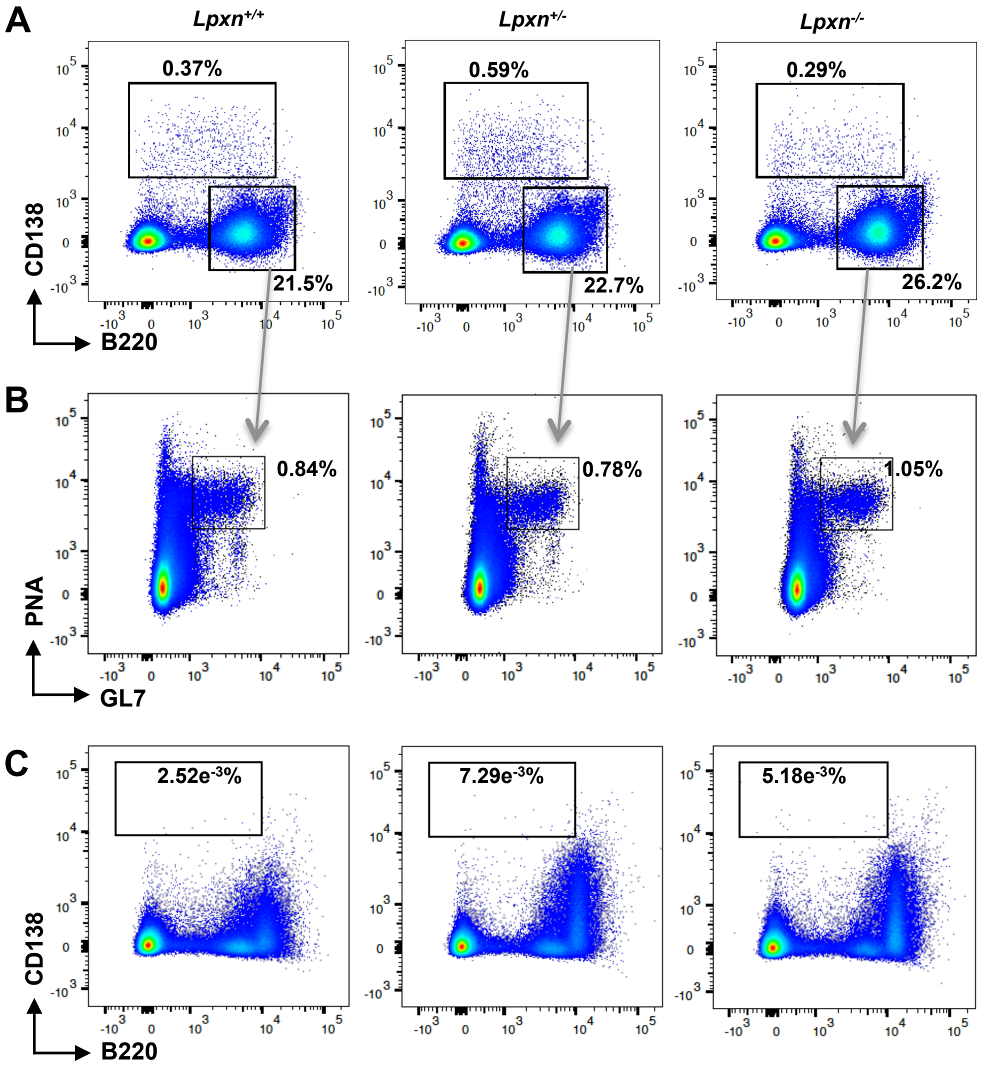

Supplementary Figure 7: **Gating strategy for spleen and bone marrow cells after immunization**. Representative gating for flow cytometry analysis of splenic B cells and PCs (top row), GC B cells (middle row) and BM PCs (bottom row). B cells were gated as $B220^{+}$, GC B cells were gated on $B220^{+}CD19^{+}$ B cells and as $GL7^{+}Fas^{+}$ and PCs were gated as $B220^{low/-}CD138^{+}$. Cells were gated on their size and structure, their viability (Live/Dead zombie aqua) and doublets were excluded.

Target	Coupling	Clone	Company	Dilution to use
Flow Cytometry				
B220	BV786/BV421/Pe Cy7	RA3-6B2	BD Biosciences	1/300
CD19	Pe-CF594	1D3	BD Biosciences	1/500
Gl7	FITC	Gl7	BD Biosciences	1/500
Fas	Pe-Cy7	Jo2	BD Biosciences	1/500
CD43	PE	S7	BD Biosciences	1/200
c-kit	FITC	2B8	BD Biosciences	1/100
IgM	APC-eFluor780	II/41	eBiosciences	1/200
CD138	BV421	281-2	BD Biosciences	1/300
NP	PE		BioCat	1/600
CD21	APC	7G6	BD Biosciences	1/200
CD23	BB700	B3B4	BD Biosciences	1/200
CD4	APC-eFluor780	RM4-5	Biolegend	1/200
CD8	BV605	53-6.7	BD Biosciences	1/150
CD44	Pe-Cy5	IM7	BD Biosciences	1/400
Gata3	Pe-Tx	L50-823	BD Biosciences	1/100
RORgt	Al647	Q31-378	BD Biosciences	1/100
Tbet	PE	O4-46	BD Biosciences	1/100
Ki67	AF700	16A8	biolegend	1/50
DAPI			Sigma	1/600
Annexin V	FITC		BD Biosciences	1/20
Live Dead	aqua zombie		eBiosciences	1/400
ELISA/ELISPOT				
IgG	uncoupled		Southern Biotech	1/1000
IgG1	HRP		Southern Biotech	1/4000
IgM	uncoupled		Southern Biotech	1/1000
IgM	HRP		Southern Biotech	1/4000
NP4	uncoupled		BioCat	1/2000
NP15	uncoupled		BioCat	1/400

Supplementary Table 1: **Antibody**. This table summarizes all the antibodies used in this study, their coupling and clone when relevant, the company from which we buy them and the dilution we used them at.

Supplementary Table 1

	Lpxn+/+	Lpxn+/-	Lpxn-/-
BM	$3.92x10^{7}$+/-$5.4x10^{6}$	$3.34x10^{7}$+/-$7.55x10^{6}$	$3.30x10^{7}$+/-$5.7x10^{6}$
spleen	$9.79x10^{7}$+/-$9.97x10^{6}$	$8.85x10^{7}$+/-$8.27x10^{6}$	$10.4x10^{7}$+/-$7.1x10^{6}$
MLN	$5.8x10^{6}$+/-$1.13x10^{6}$	$7.65x10^{6}$+/-$1.39x10^{6}$	$8.66x10^{6}$+/-$1.078x10^{6}$
PP	$1.01x10^{6}$+/-$0.25x10^{6}$	$5.2x10^{6}$+/-$1.64x10^{6}$	$1.18x10^{6}$+/-$0.24x10^{6}$
ILN	$2.1x10^{6}$+/-$0.4x10^{6}$	$2.1x10^{6}$+/-$0.35x10^{6}$	$1.8x10^{6}$+/-$0.8x10^{6}$

Supplementary Table 2: **Organ cellularity in the *Lpxn* KO mouse model.** Percentage of live cells (trypan blue positives cells were excluded) is indicated for bone marrow (2 femurs/mouse), spleen, mesenteric lymph node (MLN, 1 node), Peyer's patches (PP, 2 patches/mouse), and inguinal lymph node (ILN, 1 node). N=3-6 mice in 2 experiments. The p-values were determined with the two-tailed Mann Whitney non parametric test. All p-values were not significant.

Supplementary Table 2
